# Gender differences in subtypes of depression by first incidence and age of onset: a follow-up of the Lundby population

**DOI:** 10.1007/s00406-017-0778-x

**Published:** 2017-03-18

**Authors:** Mats Bogren, L. Brådvik, C. Holmstrand, L. Nöbbelin, C. Mattisson

**Affiliations:** Division of Psychiatry, Department of Clinical Sciences, Lund University Hospital, 221 85 Lund, Sweden

**Keywords:** Community survey, Incidence, Age of onset, Depression, Melancholia, Gender gap

## Abstract

The Lundby Study is a prospective mental health survey in a community population (*N* = 3563), in which data were collected in 4 waves of field-work between 1947 and 1997. We investigated gender differences during the follow-up in overall first incidence rates, ages of onset, and incidence by age of onset patterns, in different subtypes of depression. The overall incidence rate in females was higher than males for most subtypes of depression. However, for depression with melancholic and/or psychotic features, the overall first incidence rate did not differ significantly between the genders. The mean age of onset did not differ significantly between females and males in any of the depressive subtypes. Nevertheless, females and males had different first incidence rates by age of onset patterns for unipolar non-melancholic DSM-IV mood disorder and major depressive disorder (MDD), with a consistent gender incidence gap across all ages, but with the most conspicuous gender gap in middle age. The first incidence rates by age of onset patterns for DSM-IV MDD with melancholic and/or psychotic features did not differ significantly between the genders. The findings support that females are more prone than males to develop depression with medium severity, but no gender differences were found in melancholic and/or psychotic depression. The findings may support that unipolar non-melancholic depression and melancholic and/or psychotic depression represents different disorders. Tentative explanations for this are discussed.

## Introduction

Depressive disorders are common [1]. They are associated with increased health hazards and mortality, impact on other disorders, and are predicted to be the leading cause of disease burden by 2030 [2].

In DSM-III and DSM-IV mood disorders are defined according to a unipolar–bipolar dichotomy and major depressive disorder (MDD) is the central construct [3, 4]. Melancholia, signified by lack of mood reactivity, psychomotor retardation and diurnal variation, as defined by Schneider [5], is included as a specifier of MDD, but is not nosologically central. MDD is heterogeneous [6] and the etiology is not established [7]. Some critics argue that MDD is a watered-down entity due to the lumping together of states that have nonspecific depressive features in common [8]. The critics have suggested that melancholia should be viewed as the central mood disorder, and that bipolar disorder, depression with psychotic features, catatonic depression, puerperal depression and abnormal bereavement are instances of melancholia [8]. They argue that there is an overlap between these disorders in symptomatology, neuroendocrine variables and genetics [8]. The symptomatology of melancholia is characterised by pathological mood with unrelieved gloom and apprehension that colours cognition and self-experience, resulting in preoccupation with thoughts of worthlessness, hopelessness, guilt and suicidal ideation. About 30% of melancholia patients are psychotic [8]. Melancholia is also characterised by psychomotor change, either as retardation, agitation or reduced reactivity, and vegetative dysfunction affecting, for example, sleep, appetite and sex-drive [8].

Given the suggestion to re-establish melancholia as the core mood disorder, the epidemiology of the DSM mood disorders dichotomised into non-melancholic and melancholic disorders is interesting. However, since 1980, most community studies of mood disorders have been restricted to MDD, dysthymic disorder and bipolar disorder.

An insufficiently understood phenomenon is the preponderance of depression among females [9].

Substantive factors that may predispose differently to depression are gender-bound social roles, previous mental disorders, premorbid personality, and biological and genetic factors [10]. Gender roles may influence self-affectivity, self-esteem and the development of an externalising or internalising, e.g. ruminative, coping style, affecting vulnerability. The normative gender-role relevance of a precipitant, e.g. a stressful life event, may modulate the depressogenic response. Gender differences in prior anxiety are associated with the depression gender gap [9]. Personality dimensions, associated with depression proneness may differ. Neuroticism is associated with depression proneness and has a stronger impact in females [11], and has been suggested to be influenced by reproductive hormones, conferring greater responsiveness to emotional stressors [11]. Biological differences between the genders in stress reactivity and emotional regulation may also explain the gender gap in depression [11]. Some studies support the view of a moderately higher heritability of liability to depression in females [12]. Artefactual determinants that may explain the gender gap in depression include that females may seek help and report depressive symptoms more often, and a diagnostic bias towards women. The current concept of depression may also be more adapted to a female mode of emotional disorder presentation [10].

In a review, the pooled overall annual incidence of MDD was 2.9 per 100 persons [13], regardless of gender. Some population studies have reported non-differing depression incidence rates, and some cross-sectional studies have found the gender difference in depression to be absent or small [9]. However, most incidence studies of depression have shown significantly higher overall rates in females [14] with estimated 1.5- to 3-fold higher rates of MDD [15]. The overall incidence rate of bipolar disorder in population studies has ranged between 0.13 and 0.53 per 100 person-years at risk, without significant gender differences [14, 16], and in most cross-sectional population studies bipolar disorder has also been equal [13]. In one population study, females had a higher lifetime prevalence of both DSM-IV non-melancholic and melancholic depressive disorders [17], but in a national register study the prevalence of a first-ever ICD-10 depressive episode was about twice as high in females, but no gender differences were found in melancholic or psychotic depression [18].

A peak age of risk for first onset of major depressive episode (including bipolar depression) is estimated to range from mid-late adolescence to the early 40 s [19]. The mean or median ages of onset of DSM-III/III-R/IV MDD, or ICD-10 depression have ranged between approximately 20 and 35 years, without significant gender differences [1]. The recalled mean or median ages of onset of bipolar disorder in treated or population samples have ranged from 18 to 33 years, without significant gender differences [20]. According to an older review [21], there had been no population study on the ages of onset of non-melancholic and melancholic depressive disorder. In a more recent study in female inpatients aged 30–60 years with DSM-IV recurrent MDD, there was a minor difference in onset; the mean age of onset in the non-melancholic cases was 34.3 years and in the melancholic 36.2 years [22]. However, other findings indicate that younger age may be inversely related to melancholia [23], although in a review of studies of differences between early- and late-onset depression, no evidence of differences in psychotic symptoms or psychomotor retardation was found [24].

Although average ages of onset of depression do not differ between the genders, findings indicate that the female preponderance in depression is age specific [25], emerges in puberty [30], and increases with age up to middle life [9, 10, 25]. Some studies have shown that the gender gap in depression diminishes after the age of 50–55 [26], but others suggest that it persists [10].

The Lundby Study is an investigation of the mental health in a total population that was monitored from 1947 to 1997 [27]. The Lundby Study was initiated by Erik Essen-Möller (1901–1992) as an investigation of the distribution of personality traits, mental disorders and their possible forerunners in an ordinary, general and unselected population. The Lundby Study was originally meant to be a cross-sectional study [28], but it developed into a longitudinal investigation [27]. The original subjects recruited were everyone on the parish registers of the two adjoining rural parishes that comprised the Lundby area on 1 July 1947.

In a previous study, it has been shown that for depression, broadly defined, the female incidence rate is higher than the male [29]. In the present study, we aimed to investigate whether any gender differences have emerged during the 50-year follow-up in terms of the overall first incidence rates, average ages of first onset, or incidence rate by age of first onset patterns in different groupings of disorders, with depressive features focusing on: (1) severity of depression, (2) DSM-IV disorder subtype, and (3) non-melancholic and melancholic depression.

## Materials and methods

### Study area, population and case identification

The Lundby area surrounds a village in the south of Sweden. The study had intakes on 1 July 1947 (*N* = 2550) and 1957 (*N* = 1013), when all inhabitants including newcomers were recruited (Table 1). At inception, the subjects (*N* = 3563) were between 0 and 95 years old (median 31 years). No new subjects have been added since 1957. The subjects were followed up in field surveys, regardless of residence, in 1957, 1972 and 1997.

**Table 1:** Sources of information for case finding and attrition rate by year of field study, and alive and deceased subjects in the total Lundby population (*N* = 3563)

Year	Population	Alive subjects			Deceased subjects	
		Personal examination and outside sources	Outside sources only	Attrition	Outside sources only	Attrition
1947	2550	2520	13	17	0	0
1957	3563^a^	3260	31	19	233	20
1972	3310	2777	46	4	481	2
1997	2827	1559	82	156	1018	12

*N* number of subjects

^a^In 1957 the population enrolled in 1947 (*N* = 2550) and the new participants enrolled in 1957 (*N* = 1013) were investigated

Considerable societal changes took place during the follow-up in the rural area, including development into semi-rural/suburban character, a shift from farming to industry and service professions, and an increasing location of places of employment outside the area [27]. More than half of the population moved out from the Lundby area during the study.

The field studies generated data from semi-structured interviews, informants (e.g. relatives and nursing staff), registers and case records. Psychiatrists conducted and evaluated all four field investigations. The interviews, which contained itemised checklists of observed behaviours and subjective reports, retained the same form throughout the study. Key points in the semistructured interviews were physical and mental health, contacts with medical services, primary care, psychiatric care, somatic and mental illnesses, complaints, medication, smoking habits, alcohol and substance use and women’s health. The social situation was investigated with questions about satisfaction with life, work, the emotional climate in the family and the relationship with partner. The itemised checklists focused on observable behaviours (affective and vegetative reactions) such as tension, gloominess, torpidity and sensitivity, and questions about personality traits and habitual dispositions/symptoms (mostly related to affect) such as “Are you of a nervous disposition?”, “Do you cry easily?”, “Do you get tired easily?”, “Are you easily hurt?” and “Do you feel unjustly treated?”. Discussions were also held, and the psychiatrists wrote down their impressions. Registers included nearby hospital archives, regional registers and a national patient register. Case records included in- and outpatient records from general practitioners, somatic and psychiatric clinics.

Data permitted evaluation in 99% of the study subjects between 1947 and 1972, and 94% between 1972 and 1997 (Table 1). Attrition was lower in males (5%) compared to females (7%) during the period 1972–1997 with drop-outs more common in age groups under 50 years, varying between 6–8% in males and 7–13% in females [27].

### Diagnostic assessment

Lundby depression consists of ‘depression proper’ and ‘depression plus other symptoms’. ‘Depression proper’ includes lowered mood, feelings of low vitality, lowered enjoyment of life, lack of initiative, reduced activity, inhibition, retardation, sleep disturbances, loss of appetite and weight, anxiety and fear, reduced self-esteem, guilt feelings, and diurnal variation. Depression with the addendum ‘plus other symptoms’ refers to states that are also essentially depressive, but that, in addition to depressive symptoms, also display other symptoms, e.g. somatisation or delusions, which are consistent with depression.

Lundby depression (proper and plus) lumps together mild, reactive and atypical depressive states with profound endogenous depression with inhibition, retardation and psychosis. However, in conjunction with the diagnostic assessment, the severity was classified. Every episode with depression was scored in terms of the degree of impairment that the depression was judged to cause, in accordance with Leighton et al. [30]. A mild degree of impairment, which means that daily work is usually possible but with lowered achievement, roughly corresponds to a GAF score between 70 and 61; medium impairment is roughly 60–51; severe impairment, which practically always involves a marked reduction in functional capacity or a total inability to work, is 50–31; very severe impairment, which would include, for example, depression with retardation or delusions, is 30–1 [4, 27].

After the 1997 field investigation, consensus DSM-IV diagnoses, including mood disorder, depressive disorder, MDD, dysthymic disorder, depressive disorder not otherwise specified (NOS), bipolar depression, other mood disorder (mood disorder due to a general medical condition and substance-induced mood disorder) and adjustment disorder with depressed mood, were assessed alongside Lundby diagnoses for the period 1972–1997. The impairment degree medium was chosen as threshold for caseness. Subsequently, all first episodes with Lundby depression (proper and plus) with medium, severe or very severe impairment 1947–1972 were diagnosed according to the DSM-IV, using all available information. To study depression by severity, the threshold for caseness of Lundby depression was moved from medium to severe and very severe, respectively. To study non-melancholic and melancholic depression, groups were constructed with the aid of the melancholia concept according to Taylor and Fink [8]. Melancholic depression included MDD with melancholic and/or psychotic features and/or catatonic features, bipolar depression, puerperal depression and abnormal bereavement.

### Statistical procedures

To study the first incidence rate and age at first onset of depression, a risk sample was defined by excluding from the total population subjects who, already before intake in the study, had suffered a depression or fallen ill with schizophrenia or dementia. Incidence rates for first episodes (IR) were obtained as the number of first occurrences of a disorder in subjects aged 15 years or more divided by the total number of person-years under risk for that disorder. Female/male differences of IR and mean age of onset were tested by constructing 95% confidence intervals (CI) for the female/male IR ratios and age of onset differences, respectively [31].

## Results

### Risk sample

After excluding from the total population (*N* = 3563) the subjects who, before intake, had developed depression, schizophrenia or dementia, a sample of 3505 (female, 1707; male, 1798) subjects remained. The mean age at intake of the risk sample was 31.7 (range 0–92) in females and 31.3 (range 0–89) in males. Demographic data at intake and follow-up data of the risk sample divided into those subjects who developed depression during the follow-up and those who did not are shown in Tables 2 and 3. During the follow-up, 125 subjects (females, 76; males, 49) dropped out while they were at risk of developing depression.

**Table 2:** Demographic data at inception in subjects under risk for Lundby depression, divided into subjects who during follow-up developed Lundby depression and those who did not

	Depression cases (*N* = 432)		Non-cases (*N* = 3073)	
	Females (*N* = 267)	Males (*N* = 165)	Females (*N* = 1440)	Males (*N* = 1633)
Mean age (years)	25.4 (16.1^a^)	24.4 (17.3^a^)	31.9 (23.0^a^)	32.0 (21.8^a^)
Age range (years)	0–67	0–74	0–92	0–89
Socioeconomic status				
Blue collar	117	74	617	786
White collar	28	14	136	114
Self-employed	34	16	235	270
No information	88	61	452	463
Marital status				
Never married	130	100	704	875
Married/co-habiting	134	64	641	693
Divorced/separated	1	0	10	20
Widowed	2	1	85	45

*N* number of cases/subjects

^a^Standard deviation

**Table 3:** Follow-up data in subjects who at inception were under risk for Lundby depression

	Depression cases (*N* = 432)		Non-cases (*N* = 3073)	
	Females (*N* = 267)	Males (*N* = 165)	Females (*N* = 1440)	Males (*N* = 1633)
Moved from Lundby	157	86	748	809
Mortality	88	75	677	873
Personal examination/*N* (year)				
1947	191/192	122/123	1018/1022	1148/1170
1957	263/266	159/160	1314/1327	1484/1516
1972	255/258	146/147	1115/1132	1237/1266
1997	166/179	86/90	722/763	710/760
Register data				
Inpatient care	225	122	851	903
Outpatient care	123	78	460	574
Case records	138	85	319	360
Informants	102	65	415	478

*N* number of cases

### Incident cases

During the follow-up, 432 individuals developed a Lundby depression. The corresponding DSM-IV disorders are shown in Tables 4 and 5. Only two cases did not meet criteria for any DSM-IV mood or adjustment disorder. Melancholic mood disorder according to Taylor and Fink (*N* = 29) consisted of 24 MDD and 5 bipolar depression cases. In the MDD cases, 13 had DSM-IV melancholic features, 7 DSM-IV psychotic features, and 4 both. Two of the bipolar depression cases had both melancholic and psychotic features. No cases with catatonia, puerperal depression or pathological grief were identified.

**Table 4:** Incidence rate and female/male incidence rate ratio of Lundby depression and corresponding DSM-IV disorders

	All	Females	Males	
	IR^a^	IR^a^	IR^a^	IRR (95% CI)
Lundby depression Imp 3–5	4.07	5.23	3.00	1.74 (1.44–2.12)*
Imp 4–5	1.07	1.34	0.82	1.63 (1.13–2.36)*
Imp 5	0.10	0.11	0.10	0.10 (0.35–3.41)
DSM-IV Mood disorder	3.52	4.45	2.66	1.67 (1.36–2.06)*
Depressive disorder	3.38	4.35	2.47	1.76 (1.42–2.18)*
Major depressive disorder	2.24	2.96	1.56	1.90 (1.46–2.46)*
Dysthymic disorder	0.01	0.07	0.02	3.50 (0.39–31.32)
Depressive disorder NOS	1.00	1.16	0.84	1.38 (0.95–2.01)
Bipolar depression	0.04	0.04	0.05	0.80 (0.13–4.79)
Other mood disorder^b^	0.09	0.05	0.12	0.42 (0.11–1.61)
Non-melancholic mood disorder	3.24	4.13	2.40	1.72 (1.39–2.14)*
Melancholic mood disorder^c^	0.25	0.27	0.24	1.12 (0.54–2.33)
Adjustment disorder with depressed mood	0.47	0.66	0.28	2.36 (1.31–4.24)*

*DSM-IV* diagnostic and statistical manual of mental disorders, 4th edn. [5], *IR* incidence rate, *IRR* incidence rate ratio, *95% CI* 95% confidence interval, *Imp* impairment degree according to Leighton et al. [35], *NOS* not otherwise specified

*Statistically significant

^a^Incidence rate per 1000 person-years under risk

^b^Mood disorder due to a general medical condition and substance-induced mood disorder

^c^Taylor and Fink [10]

**Table 5:** Cases and mean age of onset of Lundby depression and corresponding DSM-IV disorders

	Females (*N* = 1707)		Males (*N* = 1798)	
	C	Age (years)	C	Age (years)
Lundby depression Imp 3–5	267	47.2 (17.2^a^)	165	46.4 (16.4^a^)
Imp 4–5	74	50.3 (17.7^a^)	47	48.3 (16.6^a^)
Imp 5	6	41.3 (16.7^a^)	6	54.0 (17.5^a^)
DSM-IV Mood disorder	230	46.9 (17.1^a^)	147	47.0 (16.4^a^)
Depressive disorder	225	46.8 (17.1^a^)	137	47.0 (16.6^a^)
Major depressive disorder	157	46.0 (15.9^a^)	88	45.4 (16.3^a^)
Dysthymic disorder	4	55.5 (16.2^a^)	1	49.0
Depressive disorder NOS	64	48.4 (19.6^a^)	48	49.9 (17.0^a^)
Bipolar depression	2	49.5 (14.8^a^)	3	36.3 (19.7^a^)
Other mood disorder^b^	3	51.7 (25.8^a^)	7	51.0 (9.5^a^)
Non-melancholic mood disorder	215	47.0 (17.3^a^)	133	46.9 (16.3^a^)
Melancholic mood disorder^c^	15	46.2 (14.2^a^)	14	47.6 (18.0^a^)
Adjustment disorder with depressed mood	37	48.8 (18.0^a^)	16	42.1 (15.5^a^)

*DSM-IV* diagnostic and statistical manual of mental disorders, 4th edn. [5], *N* number of subjects under risk for Lundby depression, *C* incident cases during follow-up of the at risk sample, *Imp* impairment degree according to Leighton et al. [35], *NOS* not otherwise specified

^a^Standard deviation

^b^Mood disorder due to a general medical condition and substance-induced mood disorder

^c^Taylor and Fink [10]

### Overall first incidence rates

The overall first incidence rates were significantly higher in females in Lundby depression, with medium and severe impairment as cut-off for caseness, DSM-IV mood disorder, depressive disorder, MDD, adjustment disorder with depressed mood, and non-melancholic mood disorder. However, there were no significant differences by gender in the overall rates of Lundby depression with very severe impairment, DSM-IV dysthymic disorder, depressive disorder NOS, bipolar depression, other mood disorder with depressive features, and melancholic mood disorder (Table 4).

### Mean age of onset

Age of first onset had a wide variation in all diagnostic subtypes in both genders (except when cases were few). In both Lundby depression with medium impairment as threshold for caseness and DSM-IV mood disorder, the age of onset ranged from 15 to 89 years in females and 15–83 years in males. In non-melancholic mood disorder, the age of onset ranged from 15 to 89 years in females and 15–83 years in males; in melancholic mood disorder the age of onset ranged from 23 to 79 years in females and 24–83 years in males. In adjustment disorder with depressed mood, the age of onset ranged from 18 to 84 years in females and 15–67 years in males. There was no significant mean age of onset differences between the genders (Table 5).

### Gender- and age-specific incidence rates

**Fig. 1:**
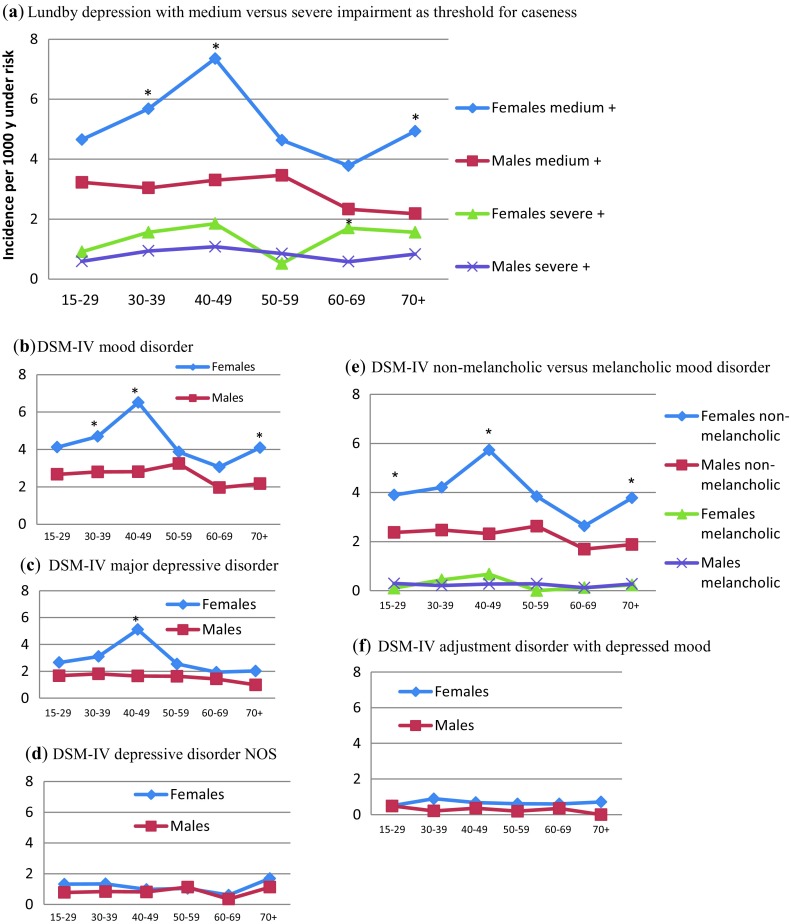
Incidence rate by age of first onset of disorders with depression in females and males grouped into: **a** Lundby depression with medium versus severe impairment as threshold for caseness; **b** DSM-IV mood disorder; **c** DSM-IV MDD; **d** DSM-IV depressive disorder NOS; **e** DSM-IV non-melancholic versus melancholic mood disorder; **f** DSM-IV adjustment disorder with depressed mood. Incidence rate per 1000 person-years under risk; age in years; medium+ corresponds to the impairment degree 3–5, and severe+ to the impairment degree 4–5 according to Leighton et al. [35]; DSM-IV, Diagnostic and Statistical Manual of Mental Disorders, 4th edn. [5]; NOS, Not Otherwise Specified; *Asterisk* statistically significant difference between females and males

Figure 1a–f shows incidence rates by age of first onset for Lundby depression, with medium and severe impairment as threshold for caseness, DSM-IV mood disorder, MDD, depressive disorder NOS, non-melancholic and melancholic mood disorder, and adjustment disorder with depressed mood. In Lundby depression with medium impairment, DSM-IV mood disorder, MDD and non-melancholic mood disorder, the female rates are consistently higher across all age bands, with a particularly distinct preponderance in the 40–49 age band. The female rates increase successively from the 15–29 to the 40–49 age bands, after which they decrease to reach a trough in the 60–69 age band; the male rates are rather stable throughout the age bands, although with a decreasing tendency in the 60–69 and 70+ age bands. In Lundby depression with severe impairment and DSM-IV depressive disorder NOS, melancholic mood disorder and adjustment disorder with depressed mood, the incidence rate by age of first onset is rather evenly distributed across the age bands in both genders and is not significantly different.

## Discussion

The aim of this study was to investigate gender differences in overall first incidence, mean age of onset and incidence by age of first onset in different subtypes of depression. Overall incidence rates of Lundby depression with medium or severe impairment, DSM-IV mood disorder, depressive disorder, MDD, non-melancholic depression and adjustment disorder with depressed mood were significantly higher in females, but no significant gender differences could be found in the overall incidence of Lundby depression with very severe impairment, DSM-IV other mood disorder with depressive features, bipolar disorder and melancholic depression. No gender differences could be found in average age of onset in any type of depression. Females across all age bands had consistently higher incidence rates of Lundby depression with medium impairment, DSM-IV mood disorder, MDD and non-melancholic mood disorder. In Lundby depression with severe impairment, DSM-IV depressive disorder NOS, melancholic depression and adjustment disorder with depressed mood age-specific rates did not differ significantly between the sexes.

### Main findings

#### Overall first incidence rate of depression

In the present study, the incidence rates of Lundby depression with medium or severe impairment as threshold for caseness, DSM-IV mood disorder, depressive disorder, MDD and adjustment disorder with depressed mood were significantly higher in females than males. This finding is in accordance with most other incidence studies. Female incidence rates of DSM-IV dysthymic disorder and depressive disorder NOS were also higher than the corresponding male rates, although not significantly. There were few cases with bipolar depression but, in line with other incidence studies, they were similar across the genders. When Lundby depression was restricted to DSM-IV other mood disorder with depressive features, the incidence rate was higher in males, but cases were too few to be conclusive. Consistent with some other studies, the gender gap in the incidence of Lundby depression declined from Lundby depression with medium impairment as threshold for caseness to Lundby depression with severe impairment as threshold for caseness; and in Lundby depression with very severe impairment as threshold for caseness, the gender gap was non-significant [18, 32].

When DSM-IV mood disorder was split into non-melancholic and melancholic disorder, the female overall incidence rate was about twice as high as the male in non-melancholic disorder. However, no significant gender incidence difference was found in melancholic disorder, a finding that contradicts one population study in which females had a higher lifetime prevalence [17], but is in accordance with the findings in a nationwide prevalence study [18].

The incidence rates of depression in the Lundby Study are low compared to most other incidence studies [13]. Methodological differences may explain this. The threshold for depression caseness may be higher in this study than in other studies, or the census method used may have lowered the incidence rates. Some of the other studies may have overestimated the first incidence rates due to recall bias, leading to underreporting of previous episodes [13, 33], or the use of structured assessments by lay interviewers may have led to artefactual inclusion of common sadness or situational unhappiness [34].

#### Average age of onset of depression

The average ages of onset of Lundby depression and the corresponding DSM-IV diagnoses were higher than previously reported. This may be because the census method biased the ages of onset upwards or because the participants in this study were frequently monitored into old age, whereas participants in some of the previous surveys were restricted to ages below 55 or 65 years [1, 35, 36]. However, we believe that the average age of onset estimates of depression in the present report may be somewhat exaggerated due to recall bias. It has previously been shown in the Lundby Study that if only participants monitored for 30 years or more are analysed, the mean age of onset of Lundby depression is 35 years [37].

In accordance with a review of previous findings [19], the age of onset of Lundby depression had a wide variation in both genders. Many cases, mainly corresponding to DSM-IV MDD, depressive disorder NOS and adjustment disorder with depressed mood, emerged in adolescence, early–middle–late adulthood and old age. The average ages of onset in the various severity and DSM-IV groupings of Lundby depression did not differ significantly between the genders. However, unlike some reports that have indicated that melancholic depression has a later onset than non-melancholic depression [22, 23], the ages of onset did not differ significantly between non-melancholic and melancholic disorder, and in both conditions the ages of onset had a wide variation across the life span. Consequently, the risk of melancholic depression did not increase with age. The mean age of onset of melancholic disorder in the Lundby population was 46.2 years in females and 47.6 in males, which was higher than in a female clinical sample [22].

#### Incidence rate by age of first onset patterns in different groupings of depression

In non-melancholic depression, the incidence rates by age of first onset were consistently higher in females across all ages, with an especially prominent female peak and gender gap in middle life, whereas in melancholic depression no incidence peak was observed and the age-specific rates were not significantly differentiated between the genders. To the best of our knowledge, this is the first time the gender-specific incidence of non-melancholic versus melancholic depression has been studied in a complete community population.

In Lundby depression with severe impairment, the incidence rates by age of onset did not differ much between the genders, although females tended to have higher rates in most age bands, including a significant difference in the 60–69 age band. However, when the incidence rate by age of onset of Lundby depression with medium impairment as threshold for caseness was analysed, the female–male rate differences were more pronounced, with a particularly prominent gender gap in middle life where only the female rate peaked. When restricting the Lundby depression cases to those meeting the criteria for DSM-IV MDD, females still retained a distinct incidence peak in the 40–49 age band, which accounted for a significant gender gap in MDD. The pattern is consistent with previous studies, which have pointed out that a gender gap in depression emerges early [38], increases with age [9, 10, 39], is particularly noticeable in middle life [25], and continues into old age [10]. The number of cases identified with DSM-IV dysthymic disorder, bipolar depression and other mood disorders with depressive features were small. This is probably due to the start in 1947, when these diagnostic concepts did not exist, so they were not accurately described in 1947, 1957 and 1972 from a DSM perspective.

### Candidate explanations of the findings

#### Premorbid personality and prior disorders

Different pathways to depression in the genders have previously been suggested in the Lundby Study, such as the pre-depressive personality traits of being easily hurt, feeling unjustly treated, tired and distracted, which bears some resemblance to a ruminative coping style that predicted Lundby depression in females. Nervous symptoms as a child and pre-depressive alcohol use disorder predicted Lundby depression in males [40]. The premorbid personality trait of being nervous and tense, and pre-depressive anxiety disorder have been shown to predict Lundby depression in both genders [40]. However, consistent with previous studies [9], anxiety disorder is more common in females in the Lundby population [41] and may partly account for the gender gap in Lundby depression and the corresponding DSM-IV depressive disorders and adjustment disorder with depressed mood.

#### Hormonal factors

In the Lundby Study, the highest incidence rate for female depression was found in the peri-menopausal age band (40–49 years), and the lowest rate in the post-menopausal age band (60–69 years). These findings are in line with previous studies showing an increased risk of developing depressive symptoms [42], including first onset of MDD [43], during the menopausal transition. Some studies have indicated that it may be the variability in the hormonal milieu in females that carries the depressogenic effect [44]. However, the association between transitional hormonal changes and depression has not yet been established [45]. We cannot directly support a hormonal model using our data since hormonal changes were not monitored in the Lundby Study, which restricts the interpretation of such a mechanism.

#### Genetic factors

Unlike non-melancholic depression, no gender gap was found in the incidence of melancholic depression in the Lundby Study. In a twin study three underlying genetic risk factor structures—cognitive/psychomotor, mood and neurovegetative—were found to reflect the DSM-IV MDD criteria [46]. The cognitive/psychomotor factor was the strongest predictor of neuroticism and comorbidity with anxiety, while the neurovegetative factor was most strongly predictive of the melancholic subtype of MDD [46]. If the cognitive/psychomotor genetic factor of MDD is overrepresented in females, and the neurovegetative genetic factor is equally represented in the genders, it might explain why there is a gender gap in non-melancholic but not melancholic depression. However, although this may be a plausible explanation, we cannot directly support it by data in the Lundby Study, which limits the interpretation.

#### Artefactual factors

Candidate artefactual factors behind the gender gap in Lundby depression and the corresponding DSM-IV depressive disorders and adjustment disorder with depressed mood are different patterns in the genders with regard to help-seeking, remembering, reporting and attrition, as well as gender-specific observation and measurement biases [27].

### Strengths and limitations

#### Strengths

The Lundby population is a total, ethnically homogeneous population that has been monitored with low attrition, which reduces the risk of selection bias. The integration of information from several sources, the consistent assessment of Lundby depression, and the 50-year follow-up, which implies that many of the study subjects passed their risk period for depression during the study, increases the case finding rate and reduces the impact of artefactual factors, compared to clinical samples.

#### Limitations

The population may not be representative of the sociodemographic structure of today’s Sweden. The small size of the study population meant that it was not representative of all depressive subtypes, and there were too few cases of DSM-IV dysthymic disorder, bipolar depression and other mood disorders with depressive features to reliably estimate incidence rates and ages of onset. The number of cases of melancholic depression was also rather low, and findings regarding this disorder must be interpreted with caution. Recall bias is a limitation, since the lengths between follow-ups were 10, 15 and 25 years and probably biased the age of onset estimates upwards, although recall bias was compensated to some extent by the external sources of information. In addition to the interviews, multiple sources, including registers and case files, from the periods between field surveys, were used to collect information, which reduced the risk of recall bias. Subjects ill at inception were not included, which may have biased the age of onset estimates upward and the incidence rates downward. The higher attrition rate among younger age groups 1972–1997 may have biased the age of onset estimates upwards and the incidence rates downwards, but the attrition was low so the effect would be limited. The Lundby Study covers a period in which different diagnostic paradigms dominated, and this has probably affected the diagnostic patterns, including the complementary data sources, during the study. This in turn probably impeded the retrospective evaluation of the original data 1947–1972 according to the DSM-IV. Precipitating factors and gender-bound social roles may have influenced the incidence of depression during the study. The Lundby population has been subjected to changes in the labour market, including females entering the work force, developments in technology, education and public health care, reduction in the cohesive power of the community, church and family, introduction of birth control, new family structures, changes in social and gender roles, and increased individualisation and freedom of choice. These changes may, via influence of precipitants and gender roles, have had an impact on the gender differences found in this study. However, due to lack of testable data regarding these issues their effect on the incidence of depression remains obscure and limits the interpretation of our results. The lack of hormonal and genetic biomarkers in the Lundby Study limits the interpretation of plausible biological mechanisms behind the findings, and we cannot draw any conclusions about suggested biological mechanisms related to gender differences in depression.

## Conclusion

The findings add to the evidence that females are more predisposed than males to develop, or be exposed to factors that cause, unipolar non-melancholic depression and adjustment disorder with depressed mood. The age-specific incidence rates indicate that females aged 15 and upward are more prone to develop unipolar non-melancholic depression than males and a considerable gender gap was found in middle life. However, no gender differences were found in overall or age-specific incidence rates for depression with melancholic and/or psychotic features (including bipolar depression). The results support the view that the average ages of onset of disorders with depressive features do not differ between the sexes and that the ages of onset of all subtypes of depression vary greatly.
